# The Inhibitory Effects of *Curcuma longa* L. Essential Oil and Curcumin on *Aspergillus flavus* Link Growth and Morphology

**DOI:** 10.1155/2013/343804

**Published:** 2013-12-03

**Authors:** Flávio Dias Ferreira, Simone Aparecida Galerani Mossini, Francine Maery Dias Ferreira, Carla Cristina Arrotéia, Christiane Luciana da Costa, Celso Vataru Nakamura, Miguel Machinski Junior

**Affiliations:** ^1^Department of Basic Health Sciences, State University of Maringa, 87020-900 Maringá, PR, Brazil; ^2^Department of Biochemistry, State University of Maringa, 87020-900 Maringá, PR, Brazil

## Abstract

The essential oil from *Curcuma longa* L. was analysed by GC/MS. The major components of the oil were ar-turmerone (33.2%), **α**-turmerone (23.5%) and **β**-turmerone (22.7%). The antifungal activities of the oil were studied with regard to *Aspergillus flavus* growth inhibition and altered morphology, as preliminary studies indicated that the essential oil from *C. longa* inhibited *Aspergillus flavus* Link aflatoxin production. The concentration of essential oil in the culture media ranged from 0.01% to 5.0% v/v, and the concentration of curcumin was 0.01–0.5% v/v. The effects on sporulation, spore viability, and fungal morphology were determined. The essential oil exhibited stronger antifungal activity than curcumin on *A. flavus*. The essential oil reduced the fungal growth in a concentration-dependent manner. *A. flavus* growth rate was reduced by *C. longa* essential oil at 0.10%, and this inhibition effect was more efficient in concentrations above 0.50%. Germination and sporulation were 100% inhibited in 0.5% oil. Scanning electron microscopy (SEM) of *A. flavus* exposed to oil showed damage to hyphae membranes and conidiophores. Because the fungus is a plant pathogen and aflatoxin producer, *C. longa* essential oil may be used in the management of host plants.

## 1. Introduction

*Aspergillus* species are widely distributed grain contaminants. They are the most common fungal species that produce mycotoxins in a wide variety of tropical and subtropical foods and feedstuffs [1, 2]. Aflatoxins, secondary metabolites of various *Aspergillus* spp., have strong hepatotoxic and carcinogenic effects and have been classified by the International Agency for Research on Cancer as Class 1 substances, that is, carcinogenic to humans [2]. Therefore, the control of fungal growth in agricultural products is necessary to reduce food-borne illness. Synthetic chemicals have been employed to control fungi in grains and foodstuffs [3, 4]. However, resistance to these compounds and secondary pests can emerge [4]. Alternatives to fungicides need to provide satisfactory aflatoxin control with low impact on the environment and on human health [3]. Natural plant extracts may be an alternative to synthetic chemical agents. Vegetal oils have been used as inhibitors of toxigenic fungi and may be safer for consumption. An increasing demand for mycotoxin-free food and commodities has been noted. Particular interest has been focused on the potential application of plant essential oils for their antimicrobial and antioxidant properties. The extracts of several edible botanicals have antifungal activity [5, 6]. Some species of *Curcuma* have been used for their colour, flavour, and preservative effect in traditional Indian curries for hundreds of years. Commercially, *Curcuma* is used as a spice, dye, and source of industrial starch [7]. The chemical constituents of turmeric, *Curcuma longa* L., have significant antimicrobial and antioxidant activity. Their fungicidal effects have been demonstrated against phytopathogenic fungi [8, 9]. This study determined the effectiveness of *Curcuma longa* L. essential oil on *Aspergillus flavus* Link growth, morphology, sporulation, and spore viability *in vitro*.

## 2. Experimental Section

### 2.1. Microorganism and Culture Conditions

The aflatoxigenic strain *Aspergillus flavus* Link (AF42) was isolated from peanut seeds and identified by physiological and morphological tests [1] at the Laboratory of Chemistry and Physiology of Microorganisms (Biochemistry Department, State University of Maringá, Maringá, PR, Brazil). The isolate was stored in silica [10] and cultured on potato dextrose agar (PDA) for seven days at 25°C in the dark [11] for the production of conidia. The conidia suspension (inoculum) was prepared by washing the cultures in sterile Tween 80 (0.01%) and counting them in a Neubauer chamber.

The solid Yeast Extract Sucrose (YES) medium [12] was prepared by adding the essential oil from *C. longa* and the curcumin standard. YES without oil or standard was used as the control medium. Tests were conducted four times, and the essential oil (0.01, 0.1, 0.25, 0.5, 1.0, 2.5, and 5.0% v/v) and curcumin standard (0.01, 0.1, 0.25, and 0.5% v/v) were added to the YES medium before inoculation. Inoculum containing 10^6^ *A. flavus* conidia was added to the YES medium control and test samples. The YES cultures were incubated at 27°C/7 d (FANEM-Model 347 G, São Paulo, Brazil).

### 2.2. Essential Oil (EO) from *Curcuma longa* L.

*Curcuma longa* rhizomes from the 2009 harvest were purchased from the Açafrão Cooperative in Mara Rosa, Brazil, at a latitude of 14°1′3′′, longitude of 49°10′30′′, and elevation of 520 m. Essential oil (EO) was extracted from 60 g of the powdered rhizomes in 500 mL of n-hexane and maintained at room temperature for 12 h with stirring. After filtering (Whatman, Maidstone, England), the EO was incubated in a rotary evaporator (Fisatom-Model 803, São Paulo, Brazil) at 60°C [8]. The essential oil was stored at 4°C and protected from light.

The chemical composition of *C. longa* EO was investigated using gas chromatography mass spectrometry (GC-MS) and nuclear magnetic resonance (NMR). The GC analysis was performed with a Thermo Electron Corporation Focus GC model under the following conditions: DB-5 capillary column (30 m × 0.32 mm × 0.50 mm); column temperature 60°C (1 min) to 180°C at 3°C/min; injector temperature, 220°C; detector temperature, 220°C; split ratio, 1 : 10; carrier gas, He; and flow rate, 1.0 mL/min. The injected volume was 1 *μ*L, which was diluted in acetone (1 : 10). The GC-MS analysis was performed using a Quadrupole Mass Spectrometer (Thermo Electron Corporation, DSQ II model) that operated at 70 eV. The identification of individual components was based on 100 comparisons of their GC retention indices on nonpolar columns and comparisons with the mass spectra of authentic standards purchased from Sigma-Aldrich [13].

For NMR, ^1^H (300.06 MHz) and ^13^C NMR (75.45 MHz) spectra were recorded in deuterated chloroform (CDCl3) solution in a Mercury-300BB spectrometer with *δ* (ppm), and spectra were compared with the CDCl3 (*δ* 7.27 for ^1^H and 77.00 for ^13^C) internal standard.

### 2.3. Chemicals

The curcumin standard was the product of *Curcuma longa* (Turmeric) and was purchased from Sigma-Aldrich (St. Louis, Mo. USA). All other solvents and reagents were analytical grade.

### 2.4. Mycelial Growth and Sporulation Measurements

The effect of EO on *A. flavus* growth and sporulation was determined by growing the fungus on YES agar in the absence (control) and presence (treatments) of EO and curcumin. The media were inoculated with a single culture at the centre of the plate. For this purpose, fungi had been previously cultured in PDA in Petri dishes, using the streaking technique [1] to produce isolated colonies. *A. flavus* was subsequently incubated at 25°C for 7 days in the dark. Each treatment was replicated on six plates. Three plates were used for sporulation measurements, and the remaining plates were used to determine growth. Sporulation measurements were performed as in Guzmán-de-Peña and Ruiz-Herrera [14] with modifications. Three agar discs (8 mm diameter) were aseptically removed from the central, intermediate, and peripheral zones of each plate using a cork borer and transferred to flasks containing sterile 0.01% Tween 80 (10 mL). The spores were estimated by counting in a Neubauer chamber. The sporulation data were recorded in spores/cm^2^ of colony. Growth was recorded as the diameter of the colony on the last day of the incubation period.

### 2.5. Germination of Spores

To evaluate the effects of *C. longa *EO on the viability of *A. flavus* spores, a microculture in dialysis membrane was assessed according to Marques et al. [15] with modifications. Spore suspensions were prepared as previously described and diluted in 0.01% Tween 80 to obtain approximately 10^6^ spores/mL. A total of 50 *μ*L of each suspension was inoculated on sterile strips of dialysis membrane (1 × 1 cm) placed on the surface of Petri plates containing YES agar without *C. longa* EO (control) or with 0.01, 0.1, 0.25, 0.5, 1.0, 2.5, or 5.0% EO and 0.01 or 0.1% curcumin; the plates were incubated at 25°C for 8 hrs. The membranes were placed on a slide, stained with lacto-phenol cotton blue, and examined under the microscope. A germinated spore was considered as such when its germ-tube was longer than half the diameter of the spore. A total of 100 spores were randomly counted on each slide, yielding a total of 300 spores per treatment. Germination was reported as the percentage of spore population and compared with the corresponding control. Four slides were sampled per treatment and control. The analysis of germ-tube morphology in each slide was performed with a biological optic photomicroscope. Germinated spores had a germ-tube of at least 50% of its size. The germination percentage was determined by the arithmetic mean for each group.

### 2.6. Morphological Studies

The same plates used for sporulation measurements were employed in the morphological studies. Samples of mycelial growth were taken at the central, intermediate, and peripheral zones of the colonies. Part of the material was stained with lactophenol cotton blue and examined under a Zeiss Axiophot light microscope, and the other part was prepared for observation in a scanning electron microscope.

For SEM, the material was prepared according to Endo et al., 2010 [16]. Samples were washed in 0.01 M phosphate-buffered saline (PBS), pH 7.2, and fixed with 2.5% glutaraldehyde (Sigma Chemical, St. Louis, MO, USA) in 0.1 M sodium cacodylate buffer (EM Sciences, Philadelphia, PA, USA). The material was applied to a poly-L-lysine-coated chip coverslip (Sigma-Aldrich, St. Louis, MO, USA) for 1 h at room temperature. The material was washed in 0.1 M sodium cacodylate buffer and dried in ethanol (50–100%). The samples were subjected to critical-point drying in CO_2_ (White Martins, Rio de Janeiro, Brazil) and sputter-coated with gold (IC-50, Shimadzu, Kyoto, Japan). The morphological characteristics of the hyphae, conidiophores, phialides, and conidia were determined with a scanning electron microscope (SEM 550 SS, Shimadzu, Kyoto, Japan) operating at 15.0 kV [14].

### 2.7. Data Analysis

To compare treatments, the Kruskal-Wallis test (nonparametric ANOVA of one factor) was followed by multiple comparisons of pairs of treatments with a 5% significance level [17]. The results were obtained by statistical program functions implemented in R [18].

## 3. Results and Discussion

The essential oil from *C. longa *has an antifungal effect on *Aspergillus flavus* Link aflatoxin production [19]. The extent of inhibition of mycotoxin production is dependent on the concentration of the essential oil used. The active compounds of turmeric, *Curcuma longa* L, are classified as nonvolatile or volatile. The major nonvolatile curcuminoids are curcumin, bisdemethoxycurcumin, and demethoxycurcumin. The volatile oil from turmeric is yellowish and stiff and commonly produces a few slightly aromatic flavours; its major compounds are ar-turmerone, turmerone, ar-curcumene, zingiberene, *α*-phellandrene, curlone, 1,8-cineol, and some other sesquiterpenes. Turmerones are the major constituents of essential oil and possess many biological activities [20]. Our investigation demonstrated that the main components of the *C. longa *essential oil were ar-turmerone (33.2%), *α*-turmerone (23.5%), and *β*-turmerone (22.7%), which had retention times of 36.32 min, 36.44 min and 37.64, respectively. These results agree with those obtained by Sharma et al. [21], Singh et al. [22], and Singh et al. [23]. The other components of the essential oil and their proportions are listed in Table 1.

The essential oil from *C. longa* has been studied for its biological activities, such as its antifungal activity [9]. Singh et al. [22] observed that the essential oil from *C. longa* at 1000 ppm (0.1%) inhibited *Fusarium moniliforme* mycelial growth. The same result occurred at 2000 ppm (0.2%) for *Aspergillus niger* and *Fusarium oxysporum*. Ferreira et al. [19] have also shown the efficacy of essential oil from *C. longa* on *A. flavus* aflatoxin production, such that the essential oil at a 0.5% concentration was able to inhibit 99.9% of the aflatoxin production. *A. flavus *growth was reduced by essential oil from *C. longa* at 0.10% (Table 2). The viability of spores was reduced at 0.10% and completely inhibited in 0.50%; viability was measured as the percentage of germinating spores produced by colonies in the presence of *C. longa* essential oil. Furthermore, the essential oil significantly reduced *A. flavus* sporulation in concentrations above 0.50%. The isolated compound, curcumin, a nonvolatile curcuminoid, was tested for the same activities. Curcumin inhibited *A. flavus* growth in 0.50% and did not inhibit *A. flavus* sporulation, and it was more efficient in preventing germination compared with the essential oil.

**Figure 1:**
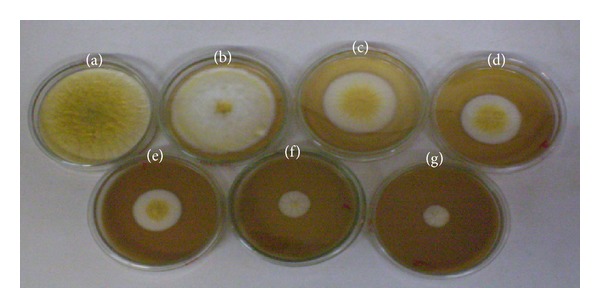
Effect of essential oil from *C. longa* on *A. flavus* mycelial growth in YES medium ((a) positive control, (b), (c), (d), (e), (f), and (g) treatments with essential oil from *C. longa* at 0.10, 0.25, 0.50, 1.0, 2.5, and 5.0%, resp.).

The results demonstrated that the active volatile compounds from turmeric, turmerones, were responsible for the inhibitory capability of the essential oil on *A. flavus* growth and morphology. The higher inhibitory effect of the essential oil compared with the isolated compound, curcumin, may also be explained by the synergistic effect of all oil components compared to a single one. Similar results were found by Vilela et al. [24], who compared the antifungal activity of the essential oil from *Eucalyptus globulus* Labill with its major component (1.8-cineol) on *A. flavus* and *A. parasiticus*. In accordance with our findings, Burt [25] suggested that the synergistic effect of minor components is important for the antimicrobial activity of essential oils. In culture media, the antifungal activity of the *C. longa* L. essential oil on *A. flavus* growth was dose-dependent (Figure 1). The antifungal activity of the essential oil from *C. longa* has been previously described [21–23]. Saju et al. [26] demonstrated that 5% *C. longa* essential oil has complete antifungal action.

Khan and Ahmad [27] demonstrated that the antifungal activity of essential oils from aromatic plants on *A. fumigatus* and *Trichophyton rubrum* was associated with the damage to the cell wall and cytoplasmic contents. Furthermore, Ultee et al. [28] demonstrated that the lipophilic properties of essential oils allow them to penetrate the plasma membrane, causing polysaccharide accumulation under drought stress conditions and leading to plasmalemma breakage in fungal cells.

**Figure 2:**
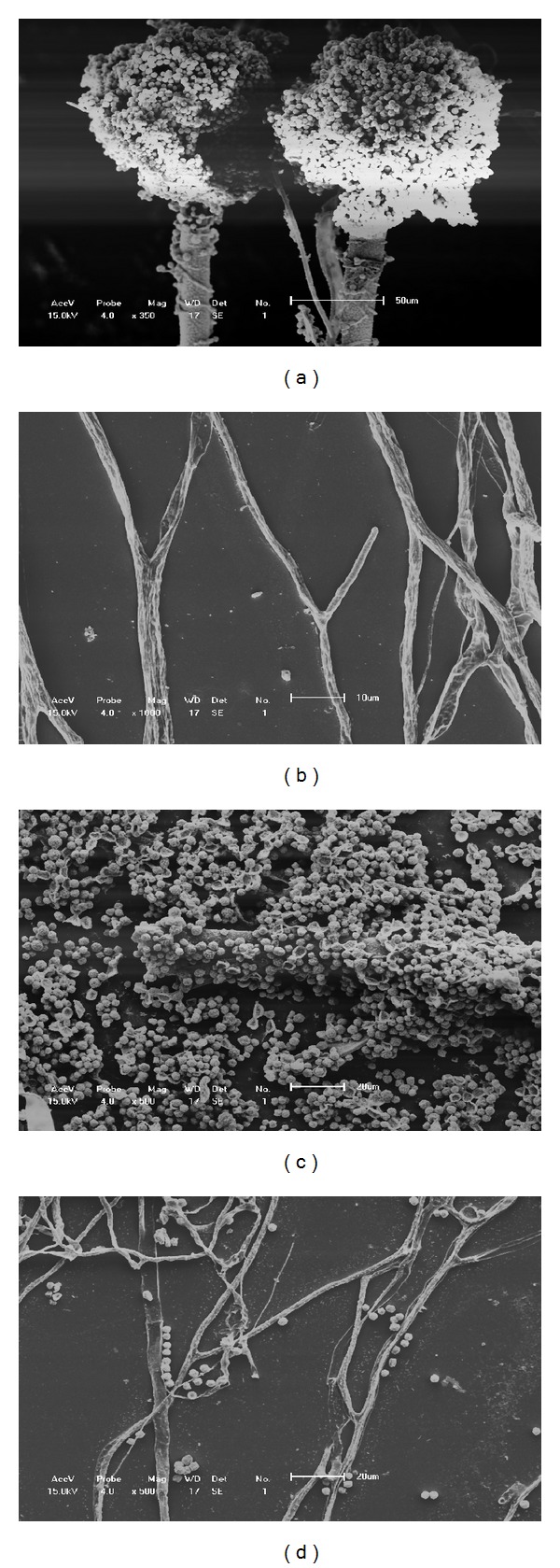
Scanning electron microscopy illustrates the effect of essential oil from *C. longa* on *A. flavus* morphology ((a), (c) positive control, (b), (d) fungi cultivated with 2.5% *C. longa* essential oil).

In addition to inhibited growth, colonies grown on solid media in the presence of any concentration of *C. longa* essential oil exhibited morphological alterations compared with the controls. Analysis by scanning electron microscopy (SEM) (Figure 2) showed the inhibition of *A. flavus* conidiophore production. Our observations showed that hyphae were targets of the oil in solid media. SEM showed that in the presence of 2.5% *C. longa* essential oil, *A. flavus* had shorter hyphae compared with the control (data not shown). Treated hyphae also showed wrinkling of the cell surface and emptying of the cytoplasmic content, which were not observed on the smooth surfaces of untreated hyphae (Figure 2). Morphological alterations did not vary in different oil concentrations. The control hyphae showed typical conidiophores (Figure 2), dichotomous branching and homogenous cytoplasm. The observed morphological characteristics were consistent with those described previously [1, 29].

The results obtained by SEM were similar to those observed after *A. niger* hyphae were treated with essential oil from *Cymbopogon nardus* [30]. Helal et al. [31] and Sharma and Tripathi [32] have also corroborated these data using essential oils from *Cymbopogon citratus* and *Citrus sinensis*, respectively. Zambonelli et al. [33] demonstrated that essential oils from *Thymus vulgaris*, *Lavandula*, and *Mentha piperita* caused the degeneration of hyphae and cytoplasmatic emptying in *Colletotrichum lindemuthianum* and *Pythium ultimum var. ultimum*. Tolouee et al. [12] reported a marked reduction in *A. niger* conidiophore formation after *Matricaria chamomilla* L. essential oil treatment.

SEM showed that the number of spores was markedly reduced in treated samples compared with the control (Figures 2(c) and 2(d)), which was confirmed by our sporulation experiment (Table 2) and a previous observation in *A. niger* treated with *Thymus* and *Thymus x-eriocalyx porlock* essential oils [34].

## 4. Conclusion

The effects of the essential oil from *Curcuma long* on the growth, spore viability, sporulation, and morphology of the aflatoxin-producing species *Aspergillus flavus* were investigated. At different concentrations, the oil reduced colony diameter, germination, and sporulation. Thus, *C. longa *EO, popularly used worldwide, may be implemented as a useful tool for controlling aflatoxin-producing fungus growth. Nevertheless, additional studies should be undertaken to determine the potential usefulness of the essential oil in fungal control programs.

## Figures and Tables

**Table 1:** Constituents of the essential oil from *Curcuma longa* L, as identified by GC/MS and RMN.

Compound	Retention index (RI)	Percentage (%)
*α*-Pinene	6.96	0.6
Vinyl propionate	7.80	1.7
P-Cymene	9.49	0.8
1.8-Cineole	10.28	0.7
Camphor	14.93	0.1
*α*-Terpineol	16.88	0.2
*β*-Caryophyllene	26.70	0.4
*γ*-Curcumene	27.74	0.5
ar-Curcumene	29.27	2.6
*α*-Zingiberene	29.76	1.0
*β*-Sesquiphellandrene	30.9	2.4
ar-Turmerol	33.08	1.5
*α*-Cadinol	35.16	1.3
ar-Turmerone	36.32	33.2
*α*-Turmerone	36.44	23.5
*β*-Turmerone	37.64	22.7
(*6R*, *7R*)-Bisabolone	40.21	3.1
(E)-*α*-Atlantone	41.51	1.4

		97.7

%: Percentage of each constituent in total essential oil.

**Table 2:** Inhibitory effect of *Curcuma  longa* L. essential oil and curcumin on *Aspergillus  flavus* Link growth, viability of spores, and sporulation *in vitro*.

	Mycelial growth		Viability of spores		Sporulation	
	Inhibition (%)	Diameter (mm)	Inhibition (%)	Germination (%)	Inhibition (%)	Spores* (×10^4^/mm^2^)
0 (control)	—	900	—	100	—	539.25
Essential oil (%)						
0.10	11.10	800^a^	53.5	46.5^a^	(—)	653.00^e^
0.25	37.45	563^a^	81.0	19.0^a^	16.64	449.50^e^
0.50	49.45	455^a^	100.0	0.0^ac^	42.88	308.00^a^
1.00	59.23	367^a^	100.0	0.0^ac^	95.78	22.75^ac^
2.50	74.34	231^ac^	100.0	0.0^ac^	98.65	7.25^ac^
5.00	77.78	200^ac^	100.0	0.0^ac^	99.67	1.75^ac^
Curcumin (%)			
0.01	(—)	900^d^	43.0	57.0^b^	(—)	578.50^d^
0.10	(—)	900^d^	88.0	12.0^b^	(—)	691.00^d^
0.25	(—)	900^d^	NR	NR	NR	NR
0.50	7.23	835^b^	NR	NR	NR	NR

Details of the experiment are described in the Experimental Section section. *Number of spores × 10^4^/mm^2^ colony. ^a,b^Values are significantly different from the control, and ^c,d,e^values do not differ among themselves, according to the multiple comparison test for nonparametric pairs of treatments at 5% significance. NR: not realized. (—) not inhibition.
